# Quantitative anatomy of the ulna’s shaft primary ossification center in the human fetus

**DOI:** 10.1007/s00276-018-2121-2

**Published:** 2018-10-31

**Authors:** Marcin Wiśniewski, Mariusz Baumgart, Magdalena Grzonkowska, Michał Szpinda, Katarzyna Pawlak-Osińska

**Affiliations:** 1 Department of Normal Anatomy, The Ludwik Rydygier Collegium Medicum in Bydgoszcz, The Nicolaus Copernicus University in Toruń, Toruń, Poland; 2 Department of Otolaryngology and Oncology, The Ludwik Rydygier Collegium Medicum in Bydgoszcz, The Nicolaus Copernicus University in Toruń, Toruń, Poland

**Keywords:** Ulna, Primary ossification center, Size, Growth dynamics, Regression analysis, Human fetuses

## Abstract

**Purpose:**

There has been little information in the medical literature regarding the growing ulna in the human fetus, though such knowledge appears to be potentially useful in diagnosing skeletal dysplasias, characterized by a disrupted or completely halted growth of the fetus. Therefore, longitudinal measurements of long bones are extremely conducive in assessing both pregnancy and fetal anatomy.

**Materials and methods:**

Using methods of CT, digital-image analysis and statistics, the size of the ulna’s shaft primary ossification center in 48 (26 males and 22 females) spontaneously aborted human fetuses aged 17–30 weeks was studied.

**Results:**

With no sex differences, the best fit growth dynamics for the ulna’s shaft primary ossification center was modeled by the following functions: *y* = − 8.476 + 1.561 × age ± 0.019 for its length, *y* = − 2.961 + 0.278 × age ± 0.016 for its proximal transverse diameter, *y* = – 0.587 + 0.107 × age ± 0.027 for its middle transverse diameter, *y* = − 2.865 + 0.226 × age ± 0.295 for its distal transverse diameter, *y* = − 50.758 + 0.251 × (age)^2^ ± 0.016 for its projection surface area, and *y* = − 821.707 + 52.578 × age ± 0.018 ± 102.944 for its volume.

**Conclusions:**

The morphometric characteristics of the ulna’s shaft primary ossification center show neither sex nor bilateral differences. The ulna’s shaft primary ossification center grows linearly with respect to its length, transverse dimensions and volume, and follows a quadratic function with respect to its projection surface area. The obtained morphometric data of the ulna’s shaft primary ossification center is considered normative for respective prenatal weeks and may be of relevance in both the estimation of fetal ages and the diagnostic process of congenital defects.

## Introduction

To date, there has been scarce information in the medical literature regarding the growing ulna in human fetuses, though such knowledge appears to be potentially useful in diagnosing skeletal dysplasias, characterized by a disrupted or completely halted growth of the fetus. Thus, longitudinal measurements of long bones are useful in assessing both pregnancy and fetal anatomy. The femoral length is most commonly and routinely measured in ultrasound examinations. However, when skeletal dysplasia is suspected, more comprehensive diagnostics is required. Either confirmation or exclusion of the preliminary diagnosis can be aided by measuring lengths of other long bones [10, 16]. The incidence of skeletal dysplasias is one per 5000 live births, which is up to 5% of children affected by congenital anomalies [9, 11]. Dysplasias in the upper limbs can affect all bones (micromelia), only the humerus (rhizomelia), the bones of the forearm (mesomelia), or the bones of the hand (acromelia). These defects are diagnosed by comparing the size of appropriate homologous bones, i.e., humerus with femur, radius with tibia, and ulna with fibula [5, 11]. Assessment of lengths of limb bones becomes an important index in the detection of osteochondrodysplasias and chromosomal abnormalities [16]. With the use of ultrasound, measuring the femoral or humeral lengths in fetuses is much easier than those of long bones in more distal body parts, as the latter are of greater mobility. Ultrasound measurements of ossified shafts of long bones are feasible from week 12 of fetal life [14], while ossification centers can be observed as early as from week 9 of fetal life [16].

Most studies concerning growth curves refer to the femur, while very few studies focus on other long bones, including the ulna. Moreover, the antebrachial and crural bones are often collectively measured, without taking each bone into account.

In the present study we aimed:

- to perform morphometric analysis of the ulna’s shaft primary ossification center in human fetuses (linear, planar and spatial parameters) in order to determine their normative values;
- to establish possible differences between sexes for all analyzed parameters;
- to compute growth dynamics for the analyzed parameters, expressed by best-matched mathematical models.

## Materials and methods

The study material comprised 48 human fetuses of both sexes (26 males and 22 females) aged 17–30 weeks, originating from spontaneous abortions and preterm deliveries. The material was acquired before the year 2000 and remains part of the specimen collection of the Department of Normal Anatomy of the Ludwik Rydygier Collegium Medicum in Bydgoszcz of the Nicolaus Copernicus University in Toruń. The experiment was approved by the Bioethics Committee of the Collegium Medicum in Bydgoszcz (KB 275/2011). The fetal age was determined based on the crown–rump length. Table 1 lists the characteristics of the study group, including age, number and sex of fetuses.

**Table 1:** Age, number and sex of the fetuses studied

Gestational age (weeks)	Crown–rump length (mm)				Number of fetuses	Sex	
	Mean	SD	Min.	Max.		♂	♀
17	116.00	1.41	115.00	117.00	2	1	1
18	130.00	0.00	130.00	130.00	2	1	1
19	150.00	3.03	146.00	154.00	6	3	3
20	159.50	0.71	159.00	160.00	2	1	1
21	174.75	2.87	171.00	178.00	4	3	1
22	184.67	1.53	183.00	186.00	3	1	2
23	197.75	2.99	195.00	202.00	4	3	1
24	208.57	3.74	204.00	213.00	7	4	3
25	214.50	0.71	214.00	215.00	2	1	1
26	226.00	1.41	225.00	227.00	2	1	1
27	237.75	2.75	235.00	241.00	4	3	1
28	246.67	4.93	241.00	250.00	3	1	2
29	254.00	1.41	253.00	255.00	2	1	1
30	263.25	1.26	262.00	265.00	4	1	3
Total					47	25	22

**Fig. 1:**
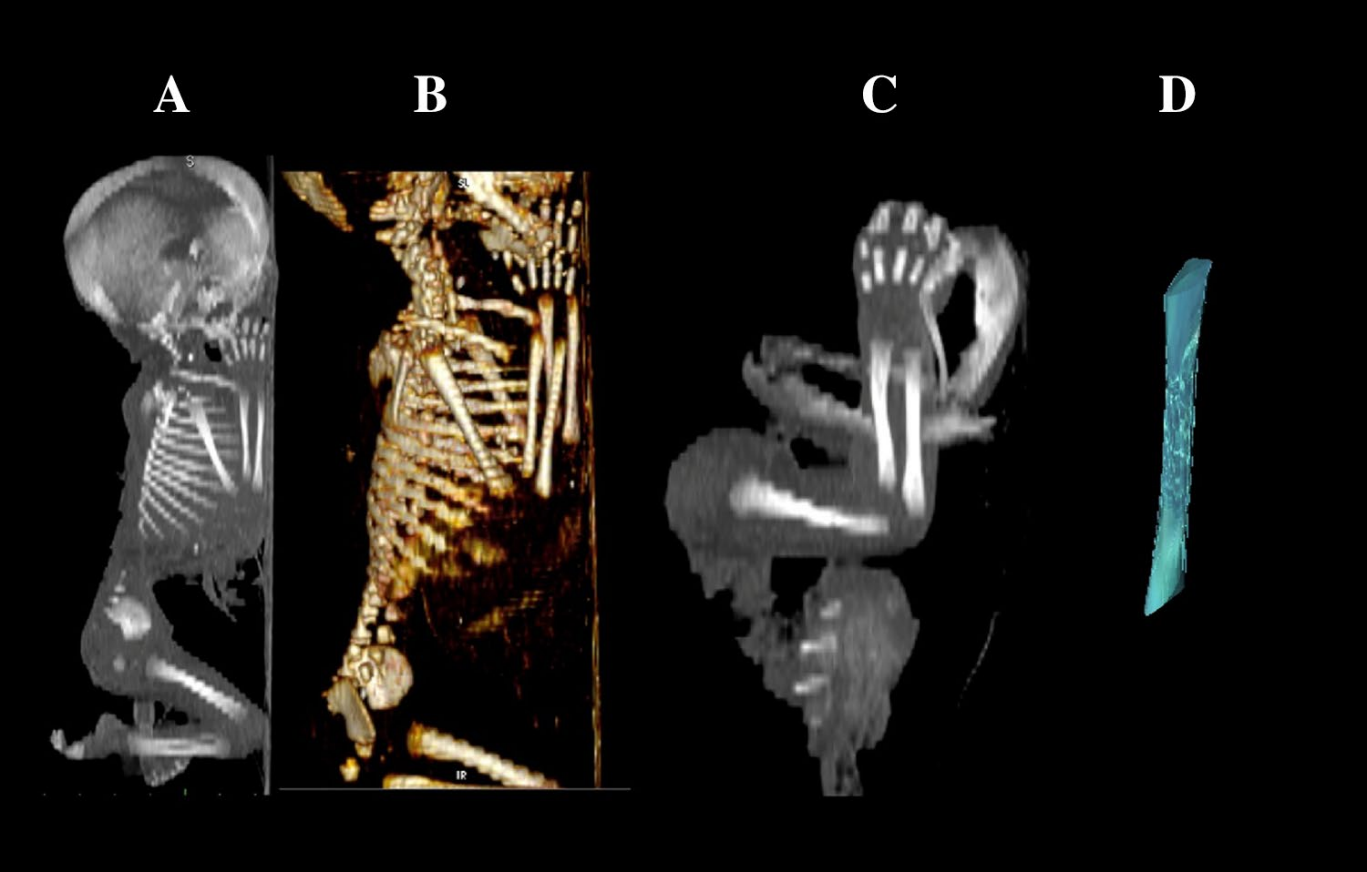
A male human fetus aged 22 weeks in the sagittal projection (**a**), its skeletal reconstruction (**b**), its right upper limb in the lateral projection (**c**), its visualization referring ulna’s shaft primary ossification center (**d**) using Osirix 3.9

Using a Siemens-Biograph 128 mCT camera (Siemens Healthcare GmbH, Erlangen, Germany) situated at Department of Positron Emission Tomography and Molecular Imaging (Oncology Center, Collegium Medicum of the Nicolaus Copernicus University, Bydgoszcz, Poland), the fetuses were scanned at a step of 0.4 mm, recorded in DICOM formats (Fig. 1), and subsequently subjected to morphometric analysis with the use of the Osirix 3.9 software. Despite the cartilaginous stage of development, contours of the proximal and distal ends of the ulna’s shaft primary ossification center were already clearly visible [8, 13], which enabled to perform its morphometric analysis regarding its transverse and sagittal dimensions, and volume.

**Fig. 2:**
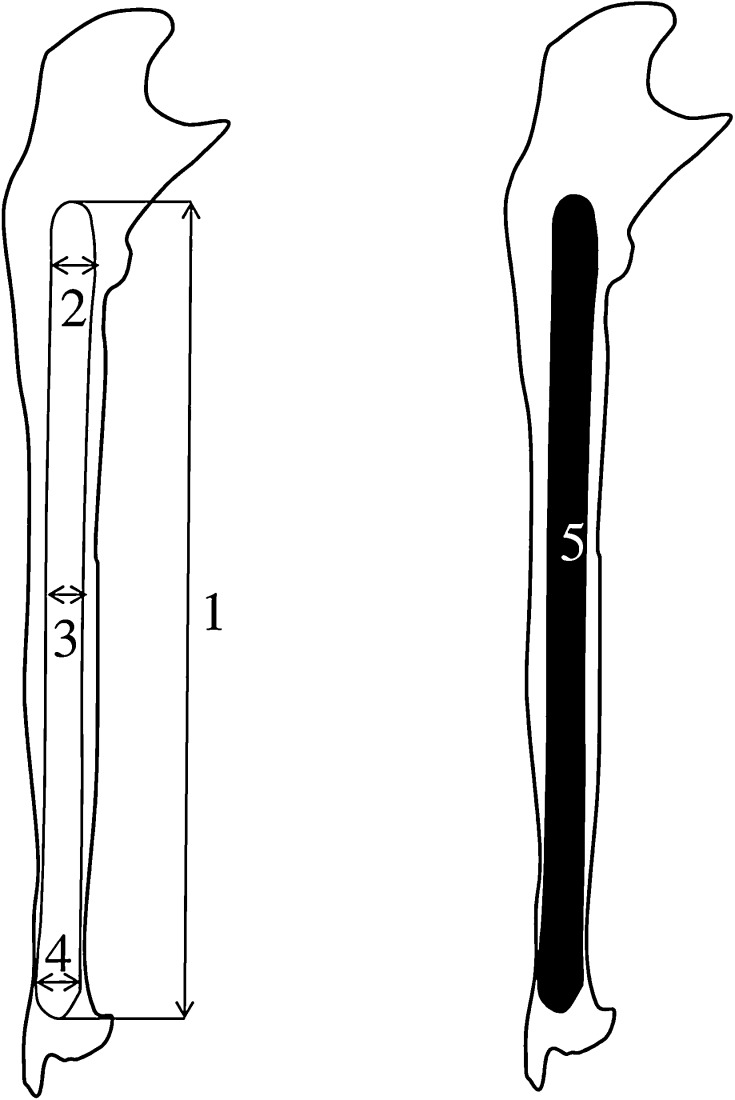
Measurement scheme of the ulna’s shaft primary ossification center in the sagittal plane. 1—length, 2—proximal transverse diameter, 3—middle transverse diameter, 4—distal transverse diameter, 5—projection surface area

Measurements of the ulna’s shaft primary ossification center were conducted in a specific order (Fig. 2). In each fetus, assessment of the linear dimensions, projection surface area and volume of the ulna’s shaft primary ossification center was carried out. Bilateral quantitative evaluation of six parameters of the ulna’s shaft primary ossification center was conducted, including:

1. length, based on the determined distance between the proximal and distal borderlines of the ossification center in the sagittal plane (Fig. 2);
2. proximal transverse diameter, based on the determined distance between the medial and lateral borderlines of the proximal region of the ossification center in the sagittal plane (Fig. 2);
3. middle transverse diameter, based on the determined distance between the medial and lateral borderlines of the central region of the ossification center in the sagittal plane (Fig. 2);
4. distal transverse diameter, based on the determined distance between the medial and lateral borderlinesof the distal region of the ossification center in the sagittal plane (Fig. 2);
5. projection surface area, based on the determined contour of the ulna’s ossification center in the sagittal plane (Fig. 2);
6. volume, calculated using advanced diagnostic imaging tools for 3D reconstruction, taking into account both position and the absorption of radiation by bone tissue (Fig. 1d).

The results achieved were statistically analyzed. Distribution of variables was checked using the Shapiro–Wilk (*W*) test, while homogeneity of variance was checked using Fisher’s test. The results were expressed as arithmetic means with standard deviations (SD). To compare the means, Student’s *t* test for independent variables and one-way analysis of variance were used. Tukey’s test was used for post hoc analysis. If no similarity of variance occurred, the non-parametric Kruskal–Wallis test was used. The characterization of developmental dynamics of the analyzed parameters was based on linear and curvilinear regression analysis. The match between the estimated curves and measurement results was evaluated based on the coefficient of determination (*R*^2^).

## Results

Mean values and standard deviations of all analyzed parameters of the right and left ulna’s shaft primary ossification centers in human fetuses at the examined age range are presented in Tables 2 and 3 for length and proximal, middle and distal transverse diameters, and in Table 4 for projection surface area and volume.

**Table 2:** Length and transverse diameters for proximal end, middle part and distal end of the right ulna’s shaft primary ossification center in human fetuses

Gestational age (weeks)	*N*	Length (mm)		Transverse diameter (mm)					
				Proximal end		Middle part		Distal end	
		Mean	SD	Mean	SD	Mean	SD	Mean	SD
17	3	18.88	0.12	1.62	0.06	1.28	0.07	1.14	0.02
18	3	19.07	0.04	1.87	0.13	1.40	0.03	1.18	0.01
19	5	19.77	0.44	2.23	0.06	1.44	0.03	1.32	0.07
20	3	22.58	0.04	2.46	0.09	1.53	0.05	1.50	0.05
21	4	24.07	1.03	2.67	0.20	1.64	0.09	1.62	0.09
22	2	27.17	0.02	3.08	0.05	1.77	0.04	1.94	0.16
23	3	27.70	0.55	3.43	0.09	1.86	0.03	2.18	0.03
24	6	28.91	0.28	3.77	0.13	1.99	0.08	2.34	0.04
25	3	30.06	0.69	4.04	0.11	2.13	0.04	2.58	0.09
26	3	31.85	0.10	4.15	0.02	2.20	0.03	2.73	0.04
27	5	32.90	0.60	4.37	0.06	2.31	0.04	2.90	0.08
28	2	34.17	0.36	4.52	0.06	2.40	0.01	3.11	0.02
29	2	34.81	0.21	4.83	0.08	2.47	0.02	3.24	0.07
30	4	37.68	0.96	5.11	0.17	2.61	0.10	3.69	0.09

**Table 3:** Length and transverse diameters for proximal end, middle part and distal end of the left ulna’s shaft primary ossification center in human fetuses

Gestational age (weeks)	*N*	Length (mm)		Transverse diameter (mm)					
				Proximal end		Middle part		Distal end	
		Mean	SD	Mean	SD	Mean	SD	Mean	SD
17	3	18.79	0.16	1.51	0.06	1.22	0.10	1.12	0.01
18	3	19.01	0.03	1.96	0.24	1.33	0.01	1.25	0.10
19	5	19.53	0.40	2.46	0.10	1.41	0.04	1.46	0.04
20	3	21.18	0.64	2.65	0.04	1.55	0.06	1.70	0.14
21	4	23.03	1.94	2.76	0.10	1.60	0.05	1.93	0.20
22	2	26.64	0.62	3.18	0.01	1.79	0.04	2.24	0.13
23	3	28.00	0.69	3.42	0.01	1.89	0.02	2.47	0.12
24	6	30.09	0.80	3.93	0.11	1.95	0.44	2.63	0.03
25	3	32.10	0.39	4.16	0.03	2.20	0.05	2.72	0.03
26	3	33.63	0.57	4.29	0.04	2.25	0.02	3.09	0.05
27	5	34.55	0.10	4.60	0.16	2.33	0.04	3.34	0.23
28	2	34.82	0.14	4.85	0.04	2.46	0.01	3.34	0.23
29	2	35.60	0.01	5.03	0.11	2.53	0.02	4.20	0.04
30	4	37.37	1.50	5.39	0.19	2.62	0.07	4.47	0.16

**Table 4:** Projection surface area and volume of the ulna’s shaft primary ossification center

Gestational age	Number of fetuses	Projection surface area (mm^2^)				Volume (mm^3^)			
		Right		Left		Right		Left	
		Mean	SD	Mean	SD	Mean	SD	Mean	SD
17	2	25.17	3.16	22.77	2.90	113.25	14.20	102.45	13.06
18	2	28.26	0.39	27.67	1.19	127.16	1.75	124.53	5.36
19	6	35.20	2.41	34.46	4.53	158.38	10.82	155.08	20.38
20	2	42.34	4.47	41.88	3.28	190.55	20.12	188.45	14.77
21	4	59.23	16.66	52.21	11.96	266.55	74.97	234.96	53.82
22	3	80.08	0.12	77.90	5.94	360.34	0.54	350.55	26.73
23	4	82.23	1.89	83.60	1.58	370.05	8.50	376.20	7.13
24	7	100.18	3.65	97.98	5.62	450.83	16.40	440.92	25.28
25	2	108.80	1.15	108.68	1.53	489.60	5.19	489.08	6.87
26	2	114.23	1.53	114.34	2.65	514.05	6.87	514.53	11.91
27	4	129.44	7.06	128.10	7.75	582.48	31.78	576.46	34.86
28	3	148.05	4.45	146.17	5.21	666.23	20.05	657.74	23.45
29	2	157.15	0.35	154.62	3.06	707.18	1.59	695.77	13.78
30	4	171.00	9.62	170.06	8.21	769.50	43.27	765.26	36.93

The statistical analysis revealed neither significant sex nor bilateral differences, which allowed us to compute one growth curve for each analyzed parameter. On both the right and left sides, the growth dynamics of the length and the three transverse diameters of the ulna’s shaft primary ossification centers followed linear functions.

**Fig. 3:**
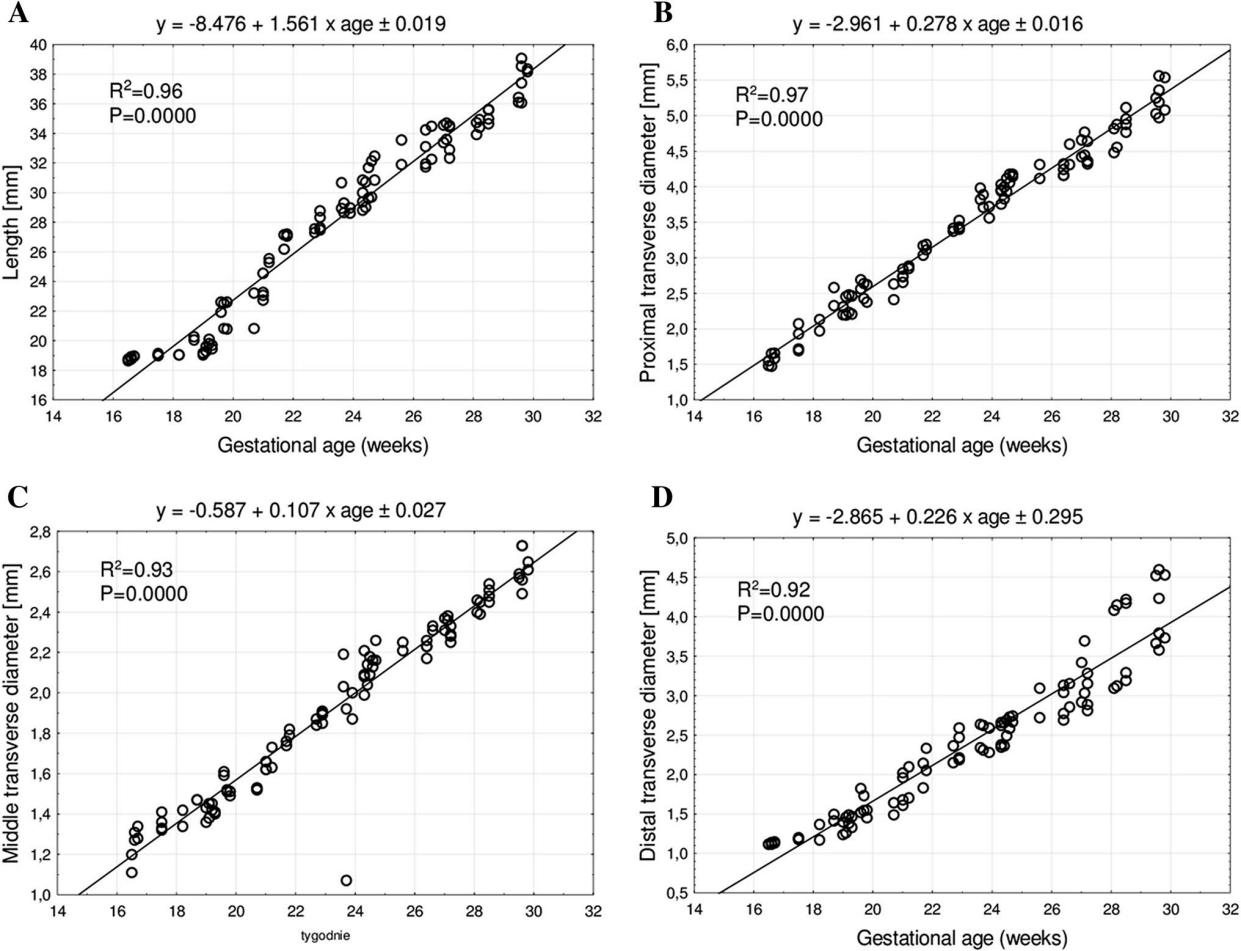
Regression lines for length (**a**), and proximal (**b**), middle (**c**) and distal (**d**) transverse diameters of the ulna’s shaft primary ossification center

The mean length of the ulna’s shaft primary ossification center in fetuses at 17–30 weeks increased from 18.88 ± 0.12 mm to 37.68 ± 0.96 mm on the right side, and from 18.79 ± 0.16 mm to 37.37 ± 1.50 mm on the left side, following the linear function *y* = − 8.476 + 1.561 × age ± 0.019 (*R*^2^ = 0.96)—(Fig. 3a).

The mean proximal transverse diameter of the ulna’s shaft primary ossification center ranged from 1.63 ± 0.06 mm at 17 weeks to 5.1 ± 0.17 mm at 30 weeks on the right side, and from 1.51 ± 0.06 mm to 5.39 ± 0.19 mm on the left side, according to the linear function *y* = − 2.961 + 0.278 × age ± 0.016 (*R*^2^ = 0.97)—(Fig. 3b). The mean middle transverse diameter of the ulna’s shaft primary ossification center at fetal ages of 17–30 weeks ranged from 1.28 ± 0.07 to 2.61 ± 0.1 mm on the right side, and from 1.22 ± 0.1 to 2.62 ± 0.07 mm on the left side, following the linear function: *y* = − 0.587 + 0.107 × age ± 0.027 (*R*^2^ = 0.93)—(Fig. 3c). The mean distal transverse diameter of the ulna’s shaft primary ossification center ranged from 1.14 ± 0.02 to 3.69 ± 0.09 mm on the right side, and from 1.12 ± 0.01 to 4.47 ± 0.16 mm on the left side, following the linear function: *y* = − 2.865 + 0.226 × age ± 0.295 (R^2^ = 0.92)—(Fig. 3d).

**Fig. 4:**
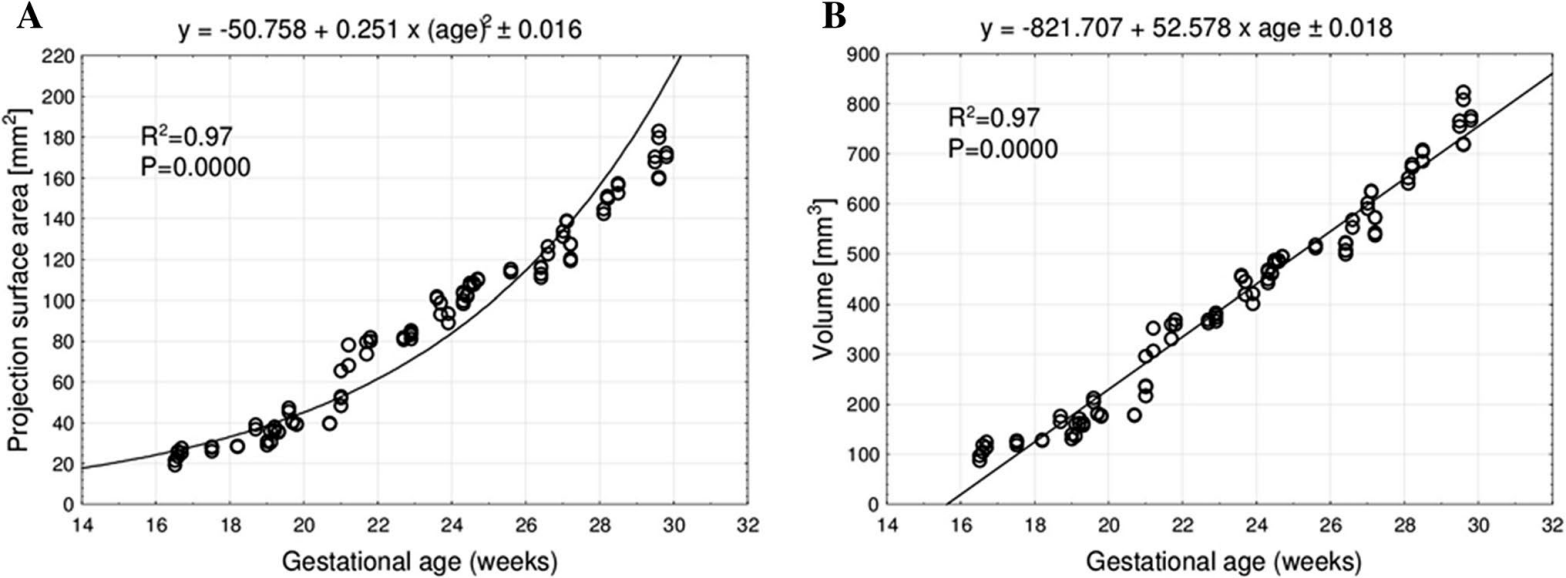
Regression lines for projection surface area (**a**), and volume (**b**) of the ulna’s shaft primary ossification center

The mean projection surface area of the ulna’s shaft primary ossification center ranged from 25.17 ± 3.16 mm^2^ at 17 weeks to 171.00 ± 9.62 mm^2^ at 30 weeks on the right side, and from 22.77 ± 0.29 to 170.06 ± 88.21 mm^2^ on the left side, following the quadratic function: *y* = − 50.758 + 0.251 × (age)^2^ ± 0.016 (*R*^2^ = 0.97)—(Fig. 4a).

The mean volume of the ulna’s shaft primary ossification center in the fetal age range of 17–30 weeks was increasing from 113.25 ± 14.20 to 769.50 ± 43.27 mm^3^ on the right side, and from 102.45 ± 13.06 to 765.26 ± 36.93 mm^3^ on the left side, following the linear function: *y* = − 821.707 + 52.578 × age ± 0.018 (*R*^2^ = 0.97)—(Fig. 4b).

## Discussion

The ossification process in the upper limb commences at the end of week 6 of fetal life. Ossification of the shafts of long bones, i.e., humerus, ulna and radius starts between weeks 7 and 12 of fetal life, while secondary ossification centers in epiphyses appear between years 1 and 3. The further bone development is mainly due to secondary ossification centers in bone condyles that appear between years 5 and 14. The development of the fetal skeletal system is most dynamic during the first trimester of pregnancy [16].

In a comprehensive ultrasound examination of 2317 pregnant women, Exacoustos et al. [14] measured lengths of long bones, including the ulna, in fetuses aged 13–40 weeks. The authors observed that between weeks 13 and 28, all measured long bones grew in a commensurate manner to fetal age, and beyond week 28 the growth followed a quadratic function of fetal age. The fastest longitudinal growth was observed for the femur, i.e., 2.8 ± 0.7 mm per week until week 28, and 1.75 ± 0.58 mm per week beyond week 28. Contrariwise, the slowest longitudinal growth was observed for the radius, i.e., 2.08 ± 0.93 mm per week until week 28, and 1.25 ± 0.75 mm per week thereafter. In turn, the ulna grew by 2.31 ± 0.85 mm per week between 13 and 28 weeks, and 1.38 ± 0.61 mm per week beyond week 28. To describe the growth of ulna, the authors generated the quadratic function of fetal age: *y*= − 31.550 + 3.709 × 0.034 (age)^2^—(R = 0.993). In the present study with fetuses aged 17–30 weeks, except for projection surface area, we observed a proportionate increase in all parameters of the ulna’s shaft primary ossification center. Its projection surface area grew in accordance with a quadratic function of fetal age.

In turn, Brons et al. [6] measured long bones in 63 fetuses aged 12–40 weeks, and demonstrated a logarithmic growth of the ulna. The length of ulna for the 50th percentile was 0.5 cm at 12 weeks and 6.4 cm at 40 weeks. Furthermore, the ulna-to-radius length index for the fiftieth percentile was roughly 1.14 throughout the examined age range. Zorzoli et al. [23] measured the length of the forearm bones, without distinguishing between the ulna and radius, in 176 fetuses aged 64–108 days. As it turned out, the bones grew according to the linear function: *y*= − 20.031 + 0.32007 × age, and the radius-to-ulna length index was 0.99 ± 0.12. Bareggi et al. [2] measured the length of long bones of the upper limb in 58 fetuses aged 8–14 weeks with CRL values between 38 and 116 mm. The authors measured the overall length of the bones and the length of their ossified parts. The overall length of the ulna in fetuses with the CRL of 38–116 mm increased from 5.5 to 31.9 mm, and besides, the ulna was longer on the right side in 30 fetuses, and on the left side in 7 fetuses. Of note, the length of its ossified part ranged from 1.8 to 26.2 mm in fetuses with the CRL of 38–116 mm. The values were greater on the right side in 24 fetuses, and on the left side in 10 fetuses. It should be noted that differences greater than 0.1 mm occurred in one case for the overall length of ulna, and in two cases for the length of the ossified part. In our study, all analyzed parameters of the ulna’s shaft primary ossification center refer to older fetuses, i.e., with the CRL of 116–265 mm, in which the length of the ossification center increased from 18.88 ± 0.12 to 37.68 ± 0.96 mm.

This paper is the first account to describe as precise as possible linear, planar and volumetric parameters of the ulna’s shaft primary ossification center and its growth dynamics in the growing human fetus. By performing morphometric analyses of different primary ossification centers in the human fetus and publishing 15 original articles in this field, we have achieved some adequate experience in image interpretation and apposite measurements. This made us assure that ossification centers assessed by CT accurately correlate well with those in “real life”.

Both the length, transverse dimensions and volume of the ulna’s shaft primary ossification center increased proportionately to fetal age, as follows: *y* = − 8.476 + 1.561 × age ± 0.019 for length, *y* = − 2.961 + 0.278 × age ± 0.016 for proximal transverse diameter, *y* = − 0.587 + 0.107 × age ± 0.027 for middle transverse diameter, *y* = − 2.865 + 0.226 × age ± 0.295 for distal transverse diameter, and *y* = − 821.707 + 52.578 × age ± 0.018 for volume. The present study also revealed the projection surface area of the ulna’s shaft primary ossification center to follow the quadratic function of fetal age in weeks: *y* = − 50.758 + 0.251 × (age)^2^ ± 0.016. It should be emphasized that the growth models of the ulna’s shaft primary ossification center were different from those of the clavicle ossification center, since the latter grew logarithmically following the functions: *y* = − 31.373 + 15.243 × ln (age) ± 1.424 with respect to transverse diameter, *y* = − 7.945 + 3.225 × ln (age) ± 0.262 with respect to proximal sagittal diameter, *y* = − 4.503 + 2.007 × ln (age) ± 0.218 with respect to middle sagittal diameter, *y* = − 4.860 + 2.117 × ln (age) ± 0.200 with respect to distal sagittal diameter, linearly with respect to its projection surface area, following the function: *y* = − 31.390 + 2.432 × age ± 4.599, and to the fourth-degree polynomial function, according to the function: y = 28.161 + 0.00017 × (age)^4^ ± 15.357 [2]. In turn, the growth dynamics of the humeral shaft ossification center followed the consecutive logarithmic functions of fetal age expressed in weeks: *y* = − 78.568 + 34.114 × ln (age) ± 2.160 for length, *y* = − 12.733 + 5.654 × ln (age) ± 0.515 for proximal transverse diameter, *y*= − 4.750 + 2.609 × ln (age) ± 0.294 middle transverse diameter, and *y* = − 10.037 + 4.648 × ln (age) ± 0.560 for distal transverse diameter. The projection surface area of the humeral shaft ossification center increased with age in a proportionate manner as *y* = − 146.601 + 11.237 × age ± 19.907, while the volume of the humeral shaft ossification center followed the fourth-degree polynomial function: *y* = 121.159 + 0.001 × (age)^4^ ± 102.944 [21].

Regrettably, we did not manage to find any reports in the medical literature concerning dimensions of the ulna’s shaft ossification center, which indubitably restricts a more comprehensive discussion in this subject. Furthermore, we failed to find any documented measurements of the ulna’s shaft primary ossification center connecting them to specific skeletal dysplasias. However, the quantitative data of the ulna’s shaft ossification center obtained in the present study may be critical in diagnosing skeletal dysplasias, frequently characterized by a disrupted or completely halted growth of the ulna. Dysplasia of the shafts of long bones, including the ulna, results in their enlargement, sclerotization, thickening of the cortical layer, and thinning or enlargement of the medullary cavity.

Achondrogenesis and thanatophoric dysplasia are lethal, with a typical image of hypoplasia of long bones of the upper limbs, including the humerus [4, 5, 11, 22].

Due to routine ultrasound examinations it is possible to diagnose developmental defects, such as skeletal dysplasias, based on reduced dimensions of long bones in relation to gestational age, and to observe abnormal morphological features and bone mineralization, as well as the presence of fractures. However, the effectiveness of this examination ranges from 40 to 60%, thus using only ultrasound is not sufficient to make a comprehensive diagnosis. When skeletal dysplasia is suspected, diagnostic imaging with the use of radiographic [15] and computed tomography [3] techniques is essential. In skeletodysplasias 3D-CT is superior to 2D-US [7, 18]. An immense advantage of the CT technique is the possibility of observing the examined structure in any plane and at any time without sacrificing image detail after examinations [21]. Compared to 2D X-ray, computed tomography eliminates an overlap of anatomical structures and allows for easy distinction between different body tissues. It is noteworthy that nowadays MRI becomes an increasingly powerful method for examining in utero fetal anatomy, especially with relation to congenital disorders of the central nervous and skeletal systems, or thoraco-abdominal organs [1]. The quality of MRI images has considerably been improved [1], mostly due to faster MRI sequences done during suspension of maternal breathing. Throughout the 2nd and 3rd trimesters of gestation, MRI may extremely be conducive when ultrasound imaging is either ambiguous or just insufficient because of a lack of an adequate acoustic window, as exemplified in oligohydramnios or breech presentation [12]. Recently, technologically advanced cine-MRI methods offer an innovative insight into movements of the whole fetus in the three-dimensional uterine environment during pregnancy [19]. Thus, the advancement of MRI techniques allows for different measurements—including those described in the present article—to be done in utero fetuses.

The main limitation of the present study has resulted from a relatively narrow fetal age, varying from 17 to 30 weeks of gestation and a somewhat small group, consisting of 48 human fetuses. Another partial limitation may be that all measurements were performed by one observer in a blind fashion. Of course, analysis of fetal CT images has some limitations, since there are some areas that require further investigation of the fetal skeleton, e.g., lengths of long bones in CT images at different gestational ages. Besides, when compared to ultrasonography, the CT evaluation of fetal bone mineralization is more difficult as there has been no standards available yet. The visualization of fetal hands and feet is also limited at earlier gestational ages, and as late as in the late second and third trimesters their images are satisfactory. Contrariwise, the advantage of fetal CT examinations results from the fact that it can be totally reinterpreted at any given time with no loss of imaging details after the study is finished [20]. Furthermore, CT examinations can discriminate one skeletal dysplasia from another in terms of impact and long-term outcome [17]. The American College of Radiology recognized a dose of less than 50 msV as no risk to the pregnant women and in utero fetus. McCollough et al. [18] even claimed that at a dose of 100 msV, the absolute risk of fetal effects was small, and at a dose of 50 msV was just negligible. To our opinion, it should be emphasized that CT examination cannot be used while evaluating minor osseous abnormalities. Instead, it may be performed as a complementary method to ultrasonography in the diagnosis of severe and potentially lethal abnormalities. As reported by Macé et al. [17], in the diagnosis of fetal skeletodysplasias, a helical CT examination is useful from week 26 of gestation and should be performed in individuals with severe micromelia below the 3rd percentile and for those under the 10th percentile associated with another bone sign. According to these authors, the fetal age above 26 weeks is a period of pregnancy which ensures additional safety, because of the development of potential exposed organs. In the third trimester of pregnancy the ossification process is satisfactory enough to correctly analyze CT images. Simultaneously, it is more difficult to obtain adequate viewing planes in three-dimensional ultrasonography.

## Conclusions

1. The morphometric characteristics of the ulna’s shaft primary ossification center show neither sex nor bilateral differences.
2. The ulna’s shaft primary ossification center grows linearly with respect to its length, transverse dimensions and volume, and follows a quadratic function with respect to its projection surface area.
3. The obtained morphometric data of the ulna’s shaft primary ossification center is considered normative for respective prenatal weeks and may be of relevance in both the estimation of fetal ages and the diagnostic process of congenital defects.
